# Development and validation of a nomogram for assessing survival in acute exacerbation of chronic obstructive pulmonary disease patients

**DOI:** 10.1186/s12890-024-03091-w

**Published:** 2024-06-19

**Authors:** Na Wang, Mengcong Li, Guangdong Wang, Lin Lv, Xiaohui Yu, Xue Cheng, Tingting Liu, Wenwen Ji, Tinghua Hu, Zhihong Shi

**Affiliations:** Department of Respiratory and Critical Care Medicine, First Affiliated Hospital of Xi’an Jiaotong University, No.277 Yanta Road, Yanta District, Xi’an, Shaanxi 710061 China

**Keywords:** AECOPD, Nomogram, Prediction, Outcome, Survival

## Abstract

**Background:**

Early prediction of survival of hospitalized acute exacerbations of chronic obstructive pulmonary disease (AECOPD) patients is vital. We aimed to establish a nomogram to predict the survival probability of AECOPD patients.

**Methods:**

Retrospectively collected data of 4601 patients hospitalized for AECOPD. These patients were randomly divided into a training and a validation cohort at a 6:4 ratio. In the training cohort, LASSO-Cox regression analysis and multivariate Cox regression analysis were utilized to identify prognostic factors for in-hospital survival of AECOPD patients. A model was established based on 3 variables and visualized by nomogram. The performance of the model was assesed by AUC, C-index, calibration curve, decision curve analysis in both cohorts.

**Results:**

Coexisting arrhythmia, invasive mechanical ventilation (IMV) usage and lower serum albumin values were found to be significantly associated with lower survival probability of AECOPD patients, and these 3 predictors were further used to establish a prediction nomogram. The C-indexes of the nomogram were 0.816 in the training cohort and 0.814 in the validation cohort. The AUC in the training cohort was 0.825 for 7-day, 0.807 for 14-day and 0.825 for 21-day survival probability, in the validation cohort this were 0.796 for 7-day, 0.831 for 14-day and 0.841 for 21-day. The calibration of the nomogram showed a good goodness-of-fit and decision curve analysis showed the net clinical benefits achievable at different risk thresholds were excellent.

**Conclusion:**

We established a nomogram based on 3 variables for predicting the survival probability of AECOPD patients. The nomogram showed good performance and was clinically useful.

**Supplementary Information:**

The online version contains supplementary material available at 10.1186/s12890-024-03091-w.

## Introduction

Acute exacerbations of chronic obstructive pulmonary disease (AECOPD) refers to the aggravation of respiratory symptoms in patients, which is the leading cause of hospitalization and medical expenditure of COPD patients [1–3], is a leading cause of substantial mortality, readmission and poor quality of life worldwide [4, 5]. In recent years, there has been a growing interest in understanding the outcomes of AECOPD and improving the management of these exacerbations. Identifying the risk factors and predict the outcome of AECOPD patients is vital and clinically useful to guide early intervention.

Studies about risk factors of outcomes of AECOPD patients found male sex, comorbidities, smoking status, the number of acute exacerbations in the previous year and abnormal laboratory findings (such as lower blood eosinophils) were associated with poor outcomes of AECOPD patients [6–9]. Recently, some studies also found new predictors of higher neutrophil-to-lymphocyte (NLR), platelet-to-lymphocyte ratio (PLR) and lymphocyte-to-monocyte ratio (LMR) were associated with outcome of AECOPD patients [10, 11].

Besides identifying risk factors, one crucial aspect of studying AECOPD outcomes is the estimation of survival rates among affected individuals. Predicting the survival of AECOPD as early as admission is help to identify people who at high risk of a poor outcome. Recently, more and more studies focus on predicting the risk of exacerbations in chronic obstructive pulmonary disease (COPD) [12, 13], and somes studies also focus on predicting the survival probability of AECOPD patients [14]. The survival probability among AECOPD patients is a critical measure of the severity and impact of these exacerbations. Understanding and emphasizing the importance of studying survival probability can help healthcare providers better assess the effectiveness of interventions, optimize treatment strategies, and ultimately improve patient outcomes. However, most of these studies only focus on one predictor, lacking of validation or sample size was small, effective models to predict the survival probability of AECOPD patients are still lacking, and value of new predictors such as NLR are also needed to be testified. Models based on multiple variables, validating and large sample size are needed.

Nomogram is based on multivariate regression analysis of multiple indicators, then represented by line segments with scores, so as to predict a certain clinical outcome or the probability of a certain type of event based on the value of multiple variables, and now well used in medical research [15, 16]. We aimed to establish a nomogram which contained multiple variables through a large sample size to predict the survival probability of AECOPD patients.

## Materials and methods

### Study population

Data of patients hospitalized for AECOPD from January 01, 2012 to December 31, 2022 were anonymized collected from Biobank of First Affiliated Hospital of Xi’an Jiaotong University with a diagnosis code of (ICD: J44.900). Diagnosis of COPD was according to GOLD 2017 criteria: Lung function tested showed forced expiratory volume in one-second/forced vital capacity (FEV1/FVC) < 0.7 after bronchodilator inhalation [17]. AECOPD was described as an acute worsening of respiratory symptoms (dyspnea, increase in sputum volume and sputum purulence) beyond, daily variation, requiring additional treatment [18].

The exclusion criteria were as follows: (1) patients aged < 40 years; (2) patients coexisting any of the following diseases: asthma, interstitial lung disease, bronchiectasis, active tuberculosis, pulmonary embolism, lung malignancy or pleural effusion; (3) patients with missing data.

### Data collection

General characteristics (age and gender), comorbidities, occurrence of respiratory failure, shock and acute kidney during hospitalization were collected from electronic medical records. Respiratory failure was determined by diagnosis on discharge records but not arterial blood gas analysis results because some patients were on oxygen at admission. Survival time refers to the time from hospital admission to date of death or discharge. Laboratory parameters at admission, including blood tests, liver and renal function, coagulation profile, cardiac enzymes and arterial blood gas analysis. NLR, PLR and LMR were calculated baesd on blood tests. Regarding treatment during hospitalization, systemic corticosteroids usage which refer to oral or injectable corticosteroids, antibiotics usage and oxygen therapy requirement were collected. The impact of systemic corticosteroid use on hematological parameters can be regarded as negligible in our study, as the hematological parameters were obtained from the initial tests conducted upon admission before patients had undergone systemic corticosteroid treatment. Intensive care stay (ICU-stay) condition, mechanical ventilation (MV) usage, which contains invasive mechanical ventilation (IMV) and noninvasive mechanical ventilation (NIMV) were also collected. The average length of hospital stay for all patients was 8.21 days. In the distribution of time to death events, there were 48 patients with outcomes within 7 days, 20 patients with outcomes between 7 and 14 days, 10 patients with outcomes between 14 and 21 days, and only 3 patients with outcomes beyond 21 days. Despite an average follow-up period of approximately 8 days in the study, with discharge of surviving patients even earlier, we observed that the data were predominantly concentrated within 7 days. Patients experiencing outcomes within 7 to 21 days accounted for approximately half of the total, while the number of patients with outcomes beyond 21 days was very limited. And considering clinical relevance, this model predicted the probability of 7-day, 14-day and 21-day survival of AECOPD patients.

### Statistical analysis

Continuous variables are presented as mean and standard deviation (SD), categorical variables are presented as frequencies and percentages (%). These patients were randomly divided into a training and a validation cohort at a 6:4 ratio, by partitioning the dataset in this ratio, we ensure that the training set has an adequate number of samples to train the model, while the validation set has sufficient samples to assess the model’s performance and generalizability. The difference between death and survival group, training cohort and validation cohort were compared, continuous variables were compared by Student’s t test, while categorical variables were compared by Chi-square test or Fisher exact test. In the training cohort, 47 prognostic factors were screened out using least absolute shrinkage and selection operator (LASSO) regression by Cox regression. LASSO is a regularization method that requires fitting only one model for each tuning parameter included in the contraction penalty term, leading to significantly enhanced computational efficiency, as well as effectively identify variables that significantly impact the target variable, thereby mitigating the effects of multicollinearity. Furthermore, it effectively identifies the optimal tuning parameter value to optimize the trade-off between bias and variance in residual sum of squares, thereby enhancing the model’s fitness for regression analysis. Additionally, LASSO can address overfitting issues by shrinking regression coefficients towards zero, thereby improving the interpretability of the model [19]. We employed multiple sampling techniques to assess the stability and generalization capabilities of the predictive model, including 10-fold cross-validation and bootstrapping. A multivariate Cox proportional hazard model were established to identify the significant prognostic factors associated with survival of AECOPD patients based on factors selected in LASSO-Cox regression, simultaneously estimate the hazard ratio (HR) and 95% confidence intervals (95% CI) of these prognostic factors, a forest map was used to visualize. Then, according to results of multivariate Cox regression analysis, factors with prognostic significance were utilized to establish a survival probability model and a nomogram was used to visualize the model. A time-dependent receiver operating characteristic (ROC) curve enables us to assess the predictive performance of the model over time, area under the ROC curve (AUC), and C-index were used to evaluate discrimination of the model in both training and validation cohorts. We fitted the model using the Cox proportional hazards model, followed by survival analysis using functions from the “survival” package in the R language. Subsequently, we calculated the survival probabilities at specific time points as needed. This approach enabled us to evaluate the model’s predictive ability for survival at different time points. The calibration plot was used to graphically evaluate the calibration of the nomogram in both training and validation cohorts. The value of the C-index ranges from 0.5 to 1.0, with 0.5 indicating random chance and closer to 1 indicates better model discrimination. The performance of the model was also evaluated with 10-fold cross-validation in the training cohort. Finally, the Decision curve analysis (DCA) of the nomogram was used to show the net clinical benefits that could be achieved under different risk thresholds in the training and validation cohorts. All analyses were conducted using R software (version 4.3.2). P-value < 0.05 (two sides) was considered statistic significant.

## Results

### Patients enrolment and establishment of training cohort and validation cohorts

**Fig. 1:**
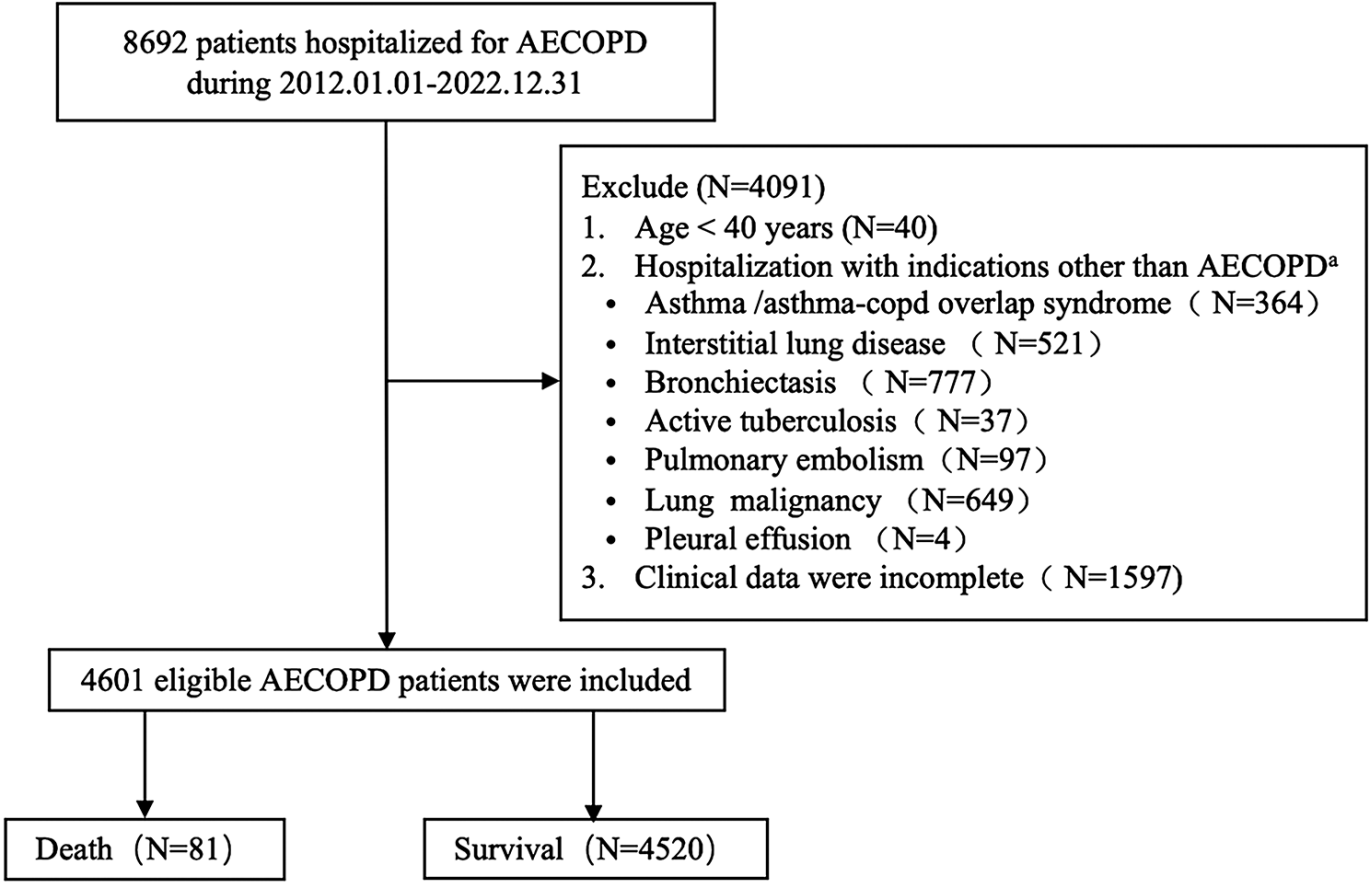
Patient enrollment flowchart. Legends: The flowchart of study population inclusion. ^a^ Including patients coexisting multiple diseases

A total of 8692 patients hospitalized with AECOPD, 4091 patients were excluded for the following reasons: (1) patients aged < 40 years (*n* = 40); (2) asthma, interstitial lung disease, bronchiectasis, active tuberculosis, pulmonary embolism, lung malignancy or pleural effusion (*n* = 2454); (3) missing data (*n* = 1597), details were shown in Fig. 1. 4601 patients were enrolled in this study finally, among whom 81 (1.77%) patients died and 4520 (98.23%) survived. 2760 patients (60%) were randomly allocated to the training cohort and 1801 (40%) to the validation cohort. There was no significant difference in most characteristics between the training cohort and validation cohort (all *P* > 0.05) (Table 1).

**Table 1:** Characteristics of the study population in the training and validation cohorts

Mean ± SD (95%CI)	Training cohort (*N* = 2760)	Validation cohort (*N* = 1841)	*P*-value
Outcome, n (%)			0.48
Survival	2715 (98.4%)	1805 (98.0%)	
Death	45 (1.63%)	36 (1.96%)	
**Gender, n (%)**			0.946
Female	665 (24.1%)	446 (24.2%)	
Male	2095 (75.9%)	1395 (75.8%)	
Age, years	71.1 (10.3)	71.4 (10.4)	0.333
Respiratory failure, n (%)	760 (27.5%)	513 (27.9%)	0.833
Shock, n (%)	21 (0.76%)	7 (0.38%)	0.152
Acute kidney injury, n (%)	14 (0.51%)	5 (0.27%)	0.324
**Comorbidities, n (%)**			
Coronary heart disease	743 (26.9%)	498 (27.1%)	0.949
Arrhythmia	483 (17.5%)	333 (18.1%)	0.637
Heart failure	92 (3.33%)	64 (3.48%)	0.997
Hypertension	897 (32.5%)	617 (33.5%)	0.493
Diabetes	337 (12.2%)	219 (11.9%)	0.784
Chronic kidney disease	67 (2.43%)	46 (2.50%)	0.956
Chronic liver disease	92 (3.33%)	64 (3.48%)	0.858
**Laboratory findings at admission**			
Red blood cell (10^12/L)	4.45(0.77)	4.44(0.78)	0.474
Hemoglobin (g/L)	136(23.0)	136(23.8)	0.818
Leukocytes (10^9/L)	7.95(5.34)	7.92(3.75)	0.826
Neutrophil (10^9/L)	6.07(3.69)	6.11(3.66)	0.715
Lymphocyte (10^9/L)	1.17(0.62)	1.17(0.66)	0.695
Eosinophil (10^9/L)	0.11(0.21)	0.11(0.19)	0.792
Platelets (10^9/L)	200(81.9)	197(81.1)	0.234
Eosinophil percentage (%)	1.70(2.66)	1.72(2.59)	0.811
NLR	7.75(11.2)	8.27(15.5)	0.218
PLR	224(184)	228(225)	0.513
LMR	3.11(3.00)	2.99(2.37)	0.132
Activated partial thromboplastin time (s)	36.9(11.2)	37.0(11.0)	0.721
Prothrombin time (s)	14.0(4.12)	13.9(3.66)	0.619
Lactose dehydrogenase (U/L)	263(457)	255(125)	0.377
Total bilirubin (umol/L)	14.2(13.1)	14.2(10.8)	0.869
Alanine aminotransferase (U/L)	30.9(67.2)	31.6(135)	0.844
Aspartate aminotransferase (U/L)	37.1(277)	34.3(139)	0.651
Albumin (g/L)	36.8(5.22)	36.6(5.55)	0.138
Uric acid (umol/L)	312(116)	304(114)	0.022^*^
Creatinine (umol/L)	69.9(43.5)	69.7(49.4)	0.927
Blood urea nitrogen (mmol/L)	6.95(3.53)	6.87(3.46)	0.451
Creatine Kinase (U/L)	96.5(227)	108(304)	0.154
CreatineKinase-MB (U/L)	15.1(15.9)	15.7(26.9)	0.380
Lacticacid, mmol/L	1.70(0.90)	1.71(0.93)	0.592
SpO2, %	91.7(8.45)	91.6(8.67)	0.696
PaO2, mmHg	74.4(27.2)	73.9(24.0)	0.508
PaCO2, mmHg	47.1(13.5)	46.8(13.2)	0.425
PH	7.40(0.06)	7.40(0.05)	0.587
**Treatment, n (%)**			
Oxygen usage	2465 (89.3%)	1668 (90.6%)	0.171
ICU-stay	702 (25.4%)	456 (24.8%)	0.635
MV usage	565 (20.5%)	378(20.5%)	0.766
NIMV usage	471 (17.1%)	312 (16.9%)	0.949
IMV usage	94 (3.41%)	66 (3.59%)	0.808
Systemic corticosteroids usage	1497 (54.2%)	1059 (57.5%)	0.03^*^
Antibiotics usage	2495 (90.4%)	1690 (91.8%)	0.117

Comparison between the the training and validation cohort

*Abbreviations*: CI: confidence interval; ICU-saty: intensive care unit stay; IMV: invasive mechanical ventilation; LMR: lymphocyte -to-monocyte ratio; MV: mechanical ventilation; NIMV: noninvasive mechanical ventilation; NLR: Neutrophil-to-Lymphocyte ratio; PaO2: arterial partial pressure of O2; PaCO2: arterial partial pressure of CO2; PLR: platelet-to-lymphocyte ratio; SpO2: arterial oxygen saturation; SD: standard deviation

^*^P < 0.05;^**^P < 0.01

### Clinical characteristics of overall enrolled AECOPD patients

The patient characteristics in the overall population were shown in Table 2. Mean age of total patients was 71.18 years, majority were male (3490; 76%), 1273 (28%) patients occured of respiratory failure during hospitalization. Nearly half of patients coexisting with heart failure (1514; 41%) followed by hypertension (33%), coronary heart disease (28%), arrhythmia (18%), diabetes (12%), chrionic liver disease (3.4%) and chronic kidney disease (2.5%). Details of laboratory findings at admission were shown in Table 2. Regarding treatment, up to 90% patients used oxygen therapy and antibiotics, more than half of patients (56%) used systemic corticosteroids, 1158 (25%) patients admitted to ICU, 943 (20%) patients required MV, among whom 783 (17%) patients required NIMV and 160 (3.5%) patients required IMV.

**Table 2:** Baseline characteristics of the study population

Mean ± SD (95%CI)	ALL patients (*N* = 4601)	Survival (*N* = 4520)	Death (*N* = 81)	*P*-value^b^
Age, years	71.18 (10.33)	71.13 (10.33)	74.23 (10.24)	0.007^**^
Male, n (%)	3490 (76%)	3427 (76%)	63 (78%)	0.7
**Death, n (%)**				
7 day			48 (59.3%)	
7–14 day			20 (24.7%)	
14-21 day			10 (12.3%)	
> 21 day			3 (3.7%)	
Length of hospitalization, days	8.21 (4.95)	8.19 (4.72)	9.62 (12.21)	0.024^*^
Cost, RMB	24,000 (34,563)	23,210 (29,949)	68,059 (126,590)	< 0.001
Respiratory failure, n (%)	1273 (28%)	1215 (27%)	58 (72%)	< 0.001
Shock, n (%)	28 (0.6%)	17 (0.4%)	11 (14%)	< 0.001
Acute kidney injury, n (%)	19 (0.4%)	14 (0.3%)	5 (6.2%)	< 0.001
**Comorbidities, n (%)**				
Coronary heart disease	1241 (27%)	1213 (27%)	28 (35%)	0.12
Arrhythmia	816 (18%)	779 (17%)	37 (46%)	< 0.001
Heart failure	1903 (41%)	1860 (41%)	43 (53%)	0.031^*^
Hypertension	1514 (33%)	1479 (33%)	35 (43%)	0.046^*^
Diabetes	556 (12%)	539 (12%)	17 (21%)	0.013^*^
Chronic kidney disease	113 (2.5%)	110 (2.4%)	3 (3.7%)	0.5
Chronic liver disease	156 (3.4%)	155 (3.4%)	1 (1.2%)	0.5
**Laboratory findings at admission**				
Red blood cell (10^12/L)	4.45 (0.78)	4.45 (0.77)	4.15 (1.10)	< 0.001
Hemoglobin (g/L)	135.71 (23.34)	135.90 (23.14)	125.21 (31.29)	< 0.001
Leukocytes (10^9/L)	7.93 (4.77)	7.91 (4.68)	9.33 (8.19)	0.4
Neutrophil (10^9/L)	6.08 (3.68)	6.06 (3.56)	7.50 (7.80)	0.2
Lymphocyte (10^9/L)	1.17 (0.64)	1.18 (0.63)	0.90 (0.67)	< 0.001
Eosinophil (10^9/L)	0.11 (0.20)	0.12 (0.20)	0.05 (0.11)	< 0.001
Platelets (10^9/L)	199.17 (81.61)	199.88 (81.30)	159.98 (89.15)	< 0.001
Eosinophil percentage (%)	1.71 (2.63)	1.73 (2.65)	0.81 (1.74)	< 0.001
NLR	7.96 (13.09)	7.81 (12.59)	16.67 (28.79)	< 0.001
PLR	225.41 (201.31)	224.15 (199.78)	295.36 (266.14)	0.063
LMR	3.06 (2.76)	3.07 (2.77)	2.62 (2.17)	0.011^*^
Activated partial thromboplastin time (s)	36.94 (11.11)	36.86 (10.90)	41.48 (18.91)	0.005^**^
Prothrombin time (s)	13.94 (3.94)	13.92 (3.94)	15.33 (3.79)	< 0.001
Lactose dehydrogenase (U/L)	259.50 (362.44)	256.71 (359.10)	414.84 (494.01)	< 0.001
Total bilirubin (umol/L)	14.21 (12.24)	14.01 (10.79)	25.20 (43.72)	0.015^*^
Alanine aminotransferase (U/L)	31.21 (100.24)	30.55 (98.70)	67.77 (161.47)	< 0.001
Aspartate aminotransferase (U/L)	36.02 (232.22)	35.39 (233.72)	71.40 (118.24)	< 0.001
Albumin (g/L)	36.71 (5.36)	36.78 (5.30)	32.37 (6.76)	< 0.001
Uric acid (umol/L)	308.96 (115.22)	307.97 (113.11)	364.37 (193.47)	0.051
Creatinine (umol/L)	69.80 (45.95)	69.23 (43.97)	101.87 (105.64)	0.032^*^
Blood urea nitrogen (mmol/L)	6.92 (3.50)	6.86 (3.39)	10.12 (6.64)	< 0.001
Creatine Kinase (U/L)	101.25 (260.62)	97.50 (223.06)	310.34 (1024.55)	0.13
CreatineKinase-MB (U/L)	15.37 (21.02)	15.12 (20.60)	29.18 (34.97)	< 0.001
Lacticacid, mmol/L	1.70 (0.91)	1.70 (0.90)	1.92 (1.37)	0.5
SpO2, %	91.66 (8.54)	91.67 (8.48)	91.27 (11.31)	0.08
PaO2, mmHg	74.23 (25.98)	74.01 (25.22)	86.43 (52.22)	0.057
PaCO2, mmHg	47.01 (13.38)	47.00 (13.29)	47.93 (17.64)	> 0.9
**Treatment, n (%)**				
Oxygen usage	4133 (90%)	4059 (90%)	74 (91%)	0.6
ICU-stay	1158 (25%)	1101 (24%)	57 (70%)	< 0.001
MV usage	943 (20%)	877 (19%)	66 (81%)	< 0.001
NIMV usage	783 (17%)	742 (16%)	41 (51%)	< 0.001
IMV usage	160 (3.5%)	135 (3.0%)	25 (31%)	< 0.001
Systemic corticosteroids usage	2556 (56%)	2493 (55%)	63 (78%)	< 0.001
Antibiotics usage	4185 (91%)	4108 (91%)	77 (95%)	0.2

Baseline characteristics of all study population and univariate analysis between the death and survival group.

Abbreviations: CI: confidence interval; ICU-saty: intensive care unit stay; IMV: invasive mechanical ventilation; LMR: lymphocyte -to-monocyte ratio; MV: mechanical ventilation; NIMV: noninvasive mechanical ventilation; NLR: Neutrophil-to-Lymphocyte ratio; PaO2: arterial partial pressure of O2; PaCO2: arterial partial pressure of CO2; PLR: platelet-to-lymphocyte ratio; SpO2: arterial oxygen saturation; SD: standard deviation. ^b^ Comparison between the death and survival group

^*^P < 0.05;^**^P < 0.01

### Comparison between death and discharge AECOPD patients

Univariate analysis between the death and survival group were showen in Table 2. The mean time from admission to death was 9.6 days. Patients who died tended to be older (74 vs. 71 years, *p* = 0.007), more patients occured of respiratory failure, shock and acute kidney injury during hospitalization (*p* < 0.001), coexisting arrhythmia, heart failure, hypertension, diabetes (*p* < 0.05). Laboratory findings included lower lymphocyte counts and platelets counts (all *p* < 0.001), higher NLR (*p* < 0.001) and LMR (*p* = 0.011), prolonged activated partial thromboplastin time and prothrombin time (*p* < 0.005), as well as higher level of lactose dehydrogenase, total bilirubin, alanine aminotransferase, aspartate aminotransferase, albumin, creatinine, blood urea nitrogen, creatine kinase and creatine kinase-MB (all *p* < 0.05). Unexpectedly, patients who died had lower eosinophil counts and eosinophil percentage (*p* < 0.001). Regarding treatment, patients who died more admitted to ICU (70% vs. 24%, *p* < 0.001), more required MV (81% vs. 19%, *p* < 0.001), both NIMV (51% vs. 16%, *p* < 0.001) and IMV (31% vs. 3%, *p* < 0.001), as well as more systemic corticosteroids usage (*p* < 0.001).

### Predict factors of AECOPD patients survival probability

**Fig. 2:**
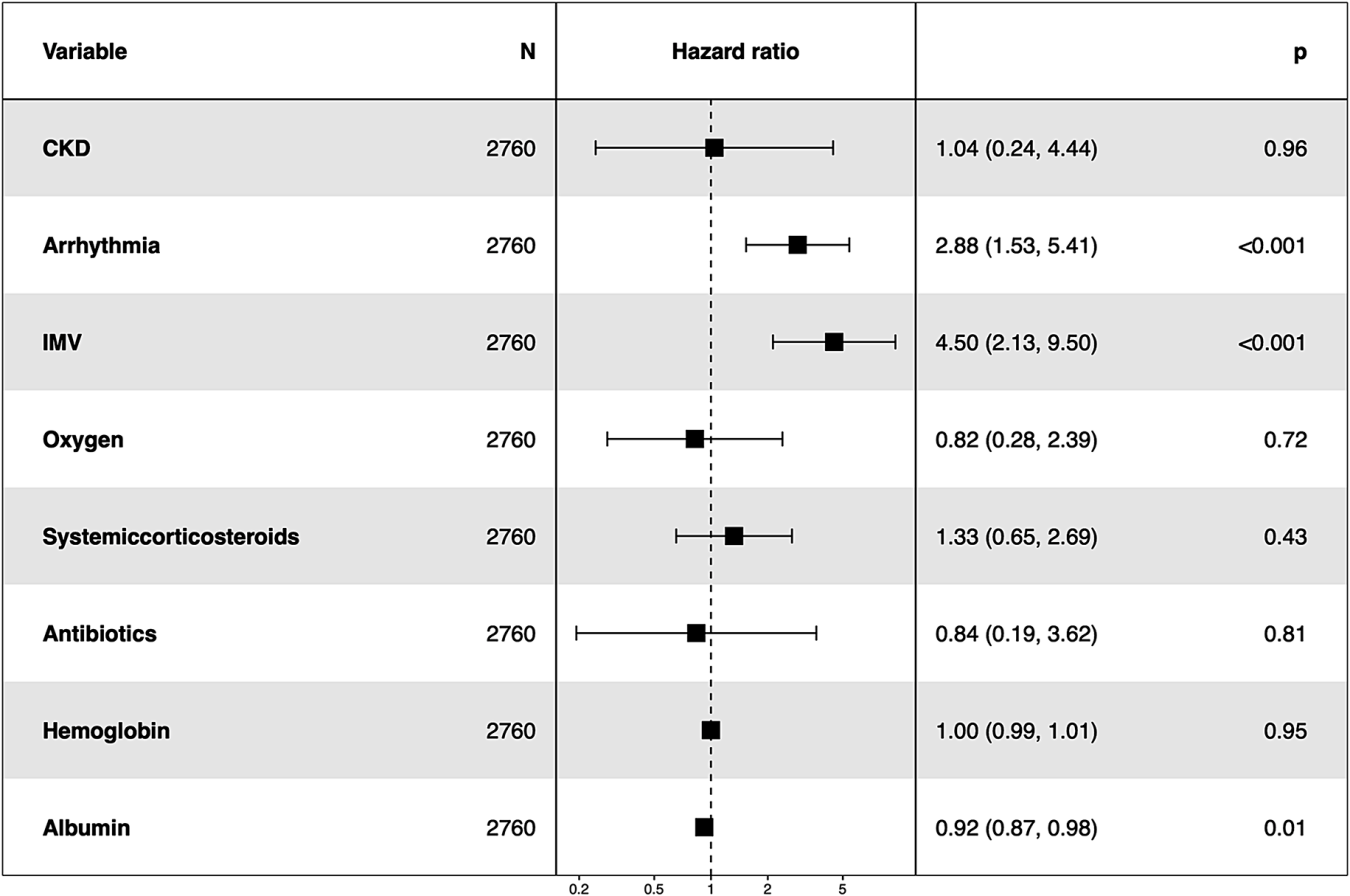
Hazard Ratios and 95% Confidence Intervals of 8 variables associated with AECOPD survival in the training cohort. Legends: The forest map of Hazard Ratios and 95% Confidence Intervals of 8 variables associated with AECOPD survival in training cohort. N indicates total number of patients in training cohort. Coexisting arrhythmia, IMV usage and lower serum albumin values were significantly associated with lower survival probability of AECOPD patients

Consideration clinical relevation and previous studies, 47 variables (listed in Table 1) from training cohort were selected as potential prognostic factors affecting survival probability and were included in LASSO-Cox regression to screen out prognostic factors which were associated with survival probability of AECOPD patients, including general characteristics, comorbidities, laboratory values and treatment. 8 variables (coexisting arrhythmia or chronic kidney disease, requiring oxygen and IMV usage, systemic corticosteroids and antibiotics usage, values of hemoglobin and albumin) were associated with survival probability when the optimal λ value was 0.07 (Supplementary Fig. S1). These 8 variables were then included in the multivariate Cox regression analyses and HR (95% CI) was shown in forest map (Fig. 2). Results showed that coexisting arrhythmia, IMV usage and lower serum albumin values were significantly associated with lower survival probability of AECOPD patients.

### Nomogram establishment and validation

**Fig. 3:**
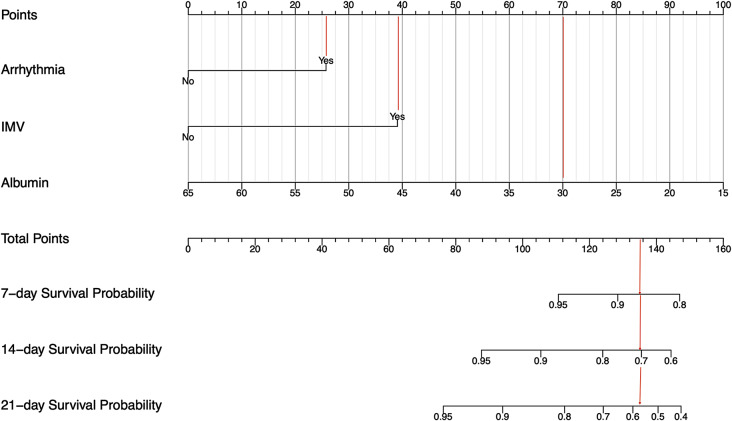
Nomogram for predicting the AECOPD patients survival probability based on training cohort. Legends: The nomogram consisting of 3 variables: arrhythmia, IMV and serum albumin values. To use the nomogram, the specific Points of individual patients are located on each variable axis. Lines and dots are drawn upward to determine the points received by each variable. The sum of these points is located on the Total Points axis. A line is drawn downward to the ‘7-day Survival Probability, 14-day Survival Probability, and 21-day Survival Probability’ axes to determine the survival probability of AECOPD patients. The unit of albumin is g/L

Coexisting arrhythmia, requiring IMV and serum albumin values were included to establish a predictive model for predicting of 7-day, 14-day and 21-day survival probability of AECOPD patients. Figure 3 shows the nomogram of the model, the usage of which is every specific value of these factors was allocated a score on the points scale, the total score was calculated by adding up these scores. Using a case example of a patient with arrhythmia requiring IMV during hospitalization, with an admission serum albumin level of 30 g/L (Fig. 3, vertical red lines). Points for arrhythmia, IMV usage, and serum albumin were 26, 39, and 70, respectively. The total points added up to 135 for this patient, which represents approximately 0.86, 0.7 and 0.57 of 7-day, 14-day and 21-day survival probability.

**Fig. 4:**
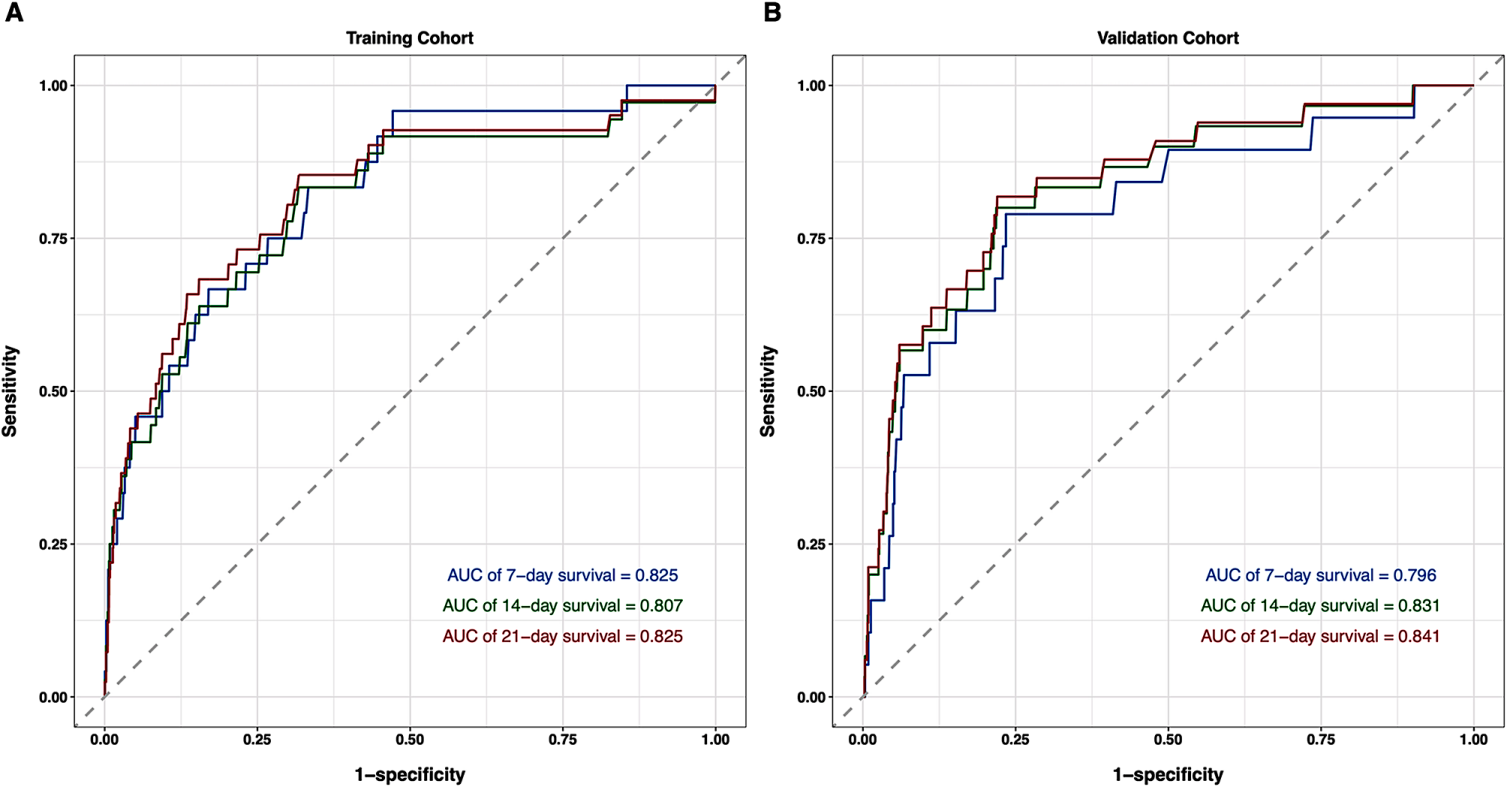
ROC curve of the nomogram in the training and validation cohort. Legends: The ROC curve and AUC of the nomogram in the training (A) and validation (B) cohort of 7-day, 14-day and 21-day survival

**Fig. 5:**
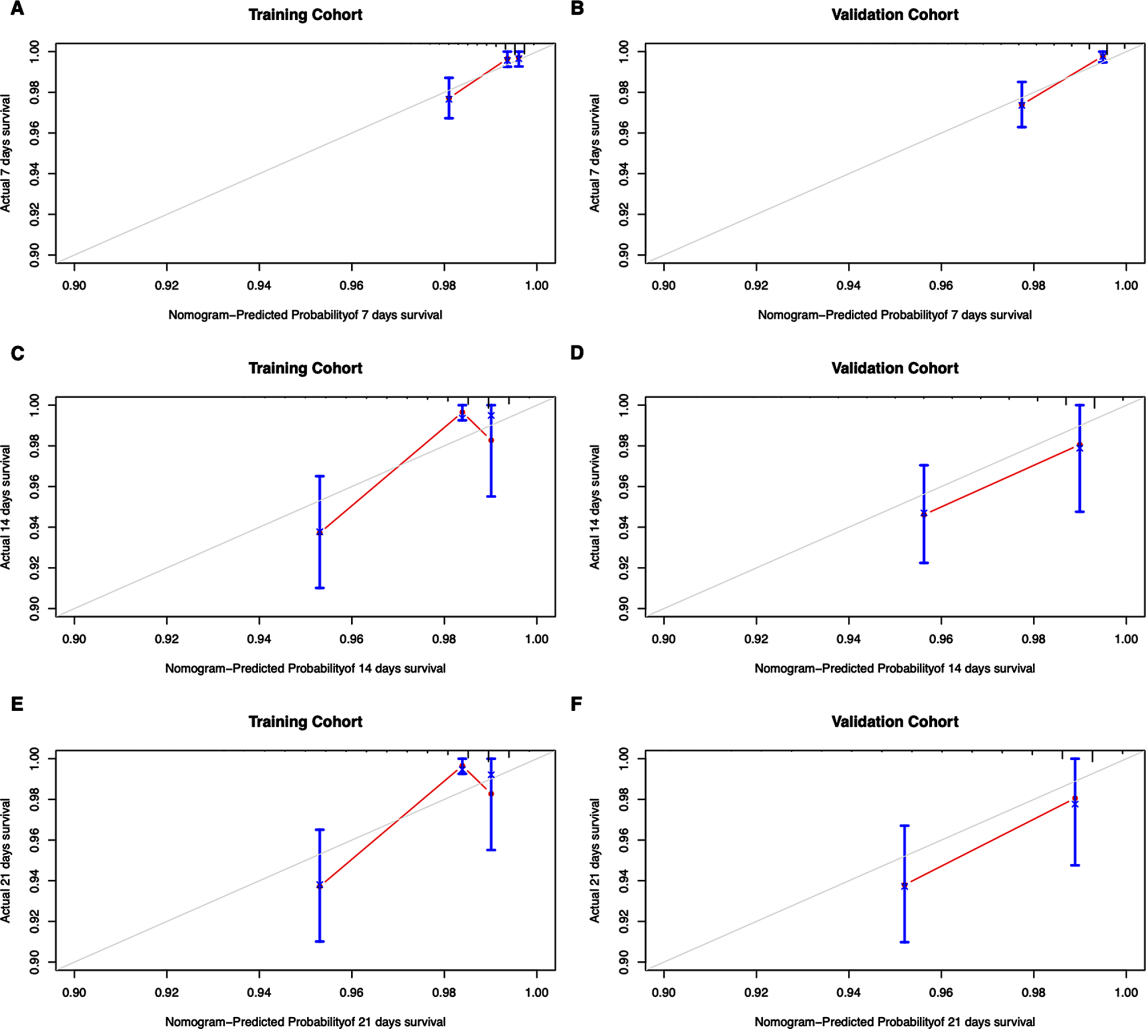
Calibration curve of the nomogram in the training and validation cohort. Legends: The calibration curve of the nomogram in the training cohort of 7-day **(A)**, 14-day **(C)** and 21-day **(E)** survival, and validation cohort of 7-day **(B)**, 14-day **(D)** and 21-day **(F)** survival. The overlap between solid and dashed lines in the line graph demonstrates the consistency between the nomogram-predicted 7-day, 14-day, and 21-day survival probabilities of AECOPD patients and the actual survival probabilities of AECOPD patients

**Fig. 6:**
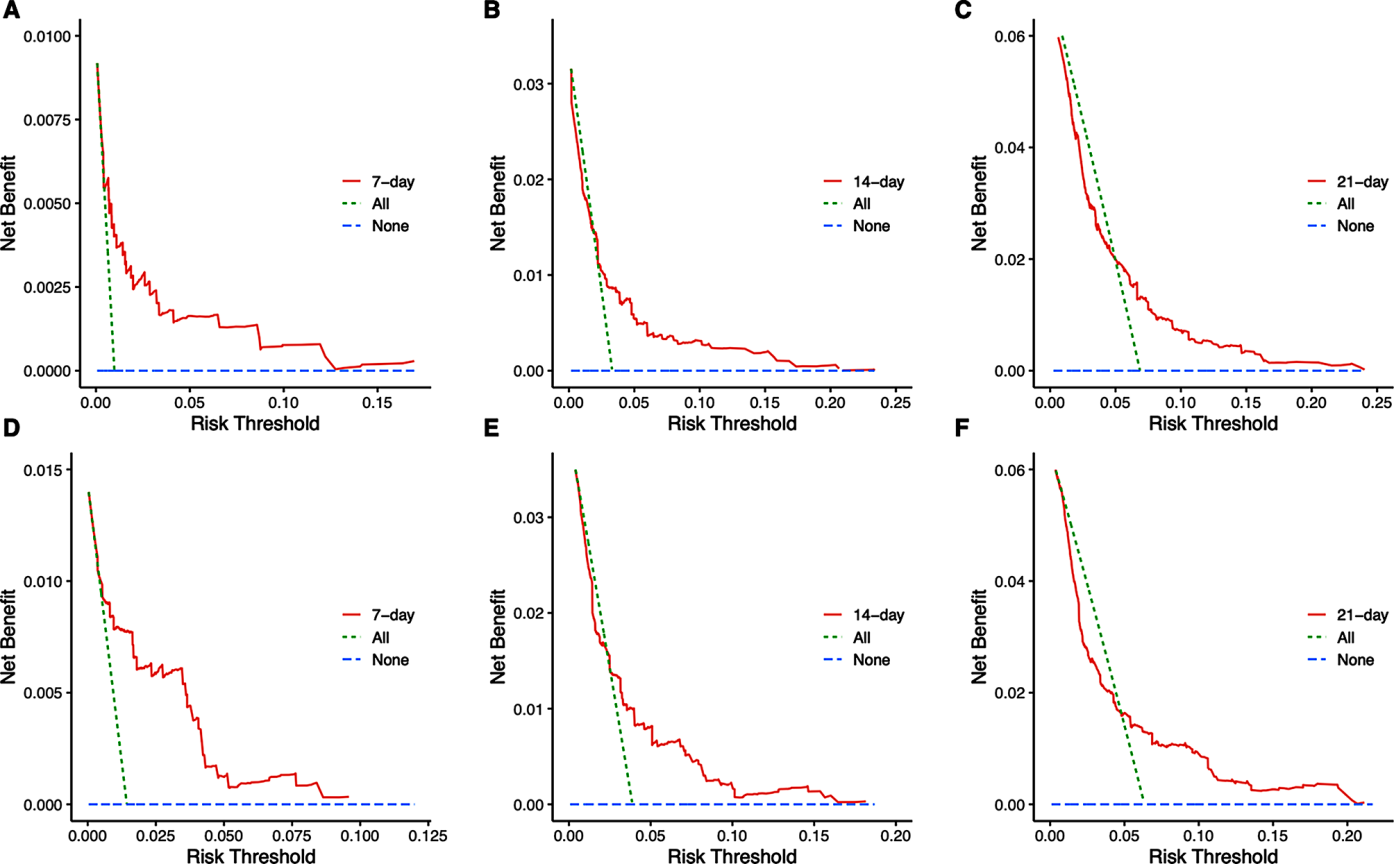
Decision curve analysis of the nomogram in the training and validation cohort. Legends: The DCA of the nomogram in the training cohort of 7-day **(A)**, 14-day**(B)** and 21-day **(C)** survival, as well as in the validation cohort of 7-day **(D)**, 14-day **(E)** and 21-day **(F)** survival. DCA depicted in the line graph illustrates the clinical net benefit achievable at various risk thresholds. The threshold range for DCA is determined based on the model’s sensitivity and specificity derived from the training and validation cohorts. Interventions are targeted towards patients within the threshold range to assess and manage risks effectively. The net benefit surpasses that of intervening for all patients or not intervening at all

The performance of discrimination ability and calibration of this nomogram in both training and validation cohorts were evaluated by C-index, AUC value and calibration curve. The C-indexes of the nomogram was 0.816 in the training cohort and 0.814 in validation cohort. The AUC in the training cohort was 0.825 for 7-day, 0.801 for 14-day and 0.825 for 21-day survival probability, and in the validation cohort this was 0.796 for 7-day, 0.831 for 14-day and 0.841 for 21-day, indicating a good discrimination ability of this model (Fig. 4). The calibration curve showed excellent agreement between the nomogram-predicted probability of survival and actual observation in training and validation cohort, which indicates good calibration of the model (Fig. 5). The DCA indicates the net clinical benefits achievable at different risk thresholds of 7-day, 14-day and 21-day in the training and validation cohort were excellent (Fig. 6).

In addition, we forced the inclusion of three factors of significance for AECOPD prognosis, including age, blood eosinophil, and leukocyte. After incorporating these three factors into the predictive model, we found that the predictive abilities of the newly included single indicators were inferior to the original predictors. Upon adding age, blood eosinophil, and leukocyte to the established model, we observed a good AUC in the training cohort, however, the C-index and AUC significantly decreased in the validation cohort. The C-index of the nomogram based on age, arrhythmia, IMV, albumin, eosinophil, and leukocyte were 0.719 in the training cohort and 0.708 in the validation cohort. The AUC in the training cohort was 0.871 for 7-day, 0.858 for 14-day and 0.851 for 21-day survival probability, in the validation cohort this was 0.779 for 7-day, 0.720 for 14-day and 0.788 for 21-day (Supplementary Table 1). Nevertheless, age, eosinophil, and leukocyte are crucial for the prognosis of AECOPD patients. We developed a predictive model incorporating these three factors, evaluated the model’s predictive ability using ROC curves (Supplementary Fig. S2), and established a nomogram (Supplementary Fig. S3).

## Discussion

Chronic obstructive pulmonary disease (COPD) is a disease state characterized by airflow limitation that is not fully reversible [17]. Patients of COPD have declined lung function, which affecting the life quality of patients seriously. AECOPD can lead to a further decline in lung function, aggravating the progression of the disease, increasing the risk of death [4]. Besides, one review of eleven studies estimated costs of exacerbations vary widely across studies from 88 to 7757 US dollars per exacerbation [20]. All of these studies revealed the importance of identifying the risk factors and predicting the outcome of AECOPD patients: guiding early intervention, improving outcomes and reducing financial burden.

LASSO regression is a regularization method for linear regression problems, which can be used to reduce the complexity of the model, prevent overfitting and select important characteristic variables. We use LASSO-Cox regression to screen for possible predictors, further multivariate Cox regression analysis showed coexisting arrhythmia, IMV usage and lower serum albumin values were significantly associated with lower survival probability of AECOPD patients for 7-day, 14-day and 21-day survival, and the model showed a good performance by assessed the C-index, AUC, and calibration plots.

Coexisting diseases such as cardiovascular disease (CVD) is common in COPD patients [21, 22]. Consistent with previous studies, our univariate analysis found died patients more coexisted with heart failure and hypertension. But alomost these studies did not include arrhythmia. One study found COPD exacerbation is associated with a high prevalence of cardiac arrhythmias [23], another study found patients with COPD are at significantly higher risk for refractory supraventricular arrhythmias. However, there is less study to investigate whether arrhythmias are associated with mortality among AECOPD patients, effect of arrhythmias on outcomes of AECOPD patients is less studied and is neglected to some extent. Arrhythmias accounted for 18% of total and was be found to be risk factor of AECOPD death in our study, which reminds us to be vigilant of arrhythmia in AECOPD patients, early identification of arrhythmia and intervention is helpful to improve prognosis of AECOPD patients.

Mechanical ventilation (MV) is helpful for patients to overcome respiratory failure caused by underlying diseases and create conditions for the treatment of underlying diseases. Invasive mechanical ventilation (IMV) is the primary choice of treatment for 5.9–8.7% of AECOPD patients [24]. In our study, there was 943 (20%) patients used IMV totally, and death patients were 2.6-folds using IMV than survivors. Indeed, death patients were in more worse situation such as more comorbidities and more ccurrence of respiratory failure in our study. Our study suggested that patients who using IMV are more likely to suffer a poorer prognosis compared with those who do not use IMV, poor condition of patients should be improved early, and more attention should be paid to AECOPD who use IMV to improve the prognosis.

Albumin is the most important protein in human plasma, maintaining the body’s nutrition and osmotic pressure, is a biomarker for nutritional status of body. Malnutrition is a consequence of reduced nutritional intake and muscle loss. Previous studies have found that death AECOPD patients had poorer nutritional status than survivors [25, 26]. Consistent with previous studies, we found patients who died having lower serum albumin level, which also indicating poorer nutritional status. COPD is a chronic inflammatory lung disorder and often combined with digestion and absorption dysfunction and high energy consumption, causing the COPD patients to suffer from malnutrition, especially in the acute exacerbation of COPD [27]. AECOPD is aggravation of respiratory symptoms in patients, and systemic inflammatory response is also aggravated, which contribute to a decrease in the albumin levels in serum [28, 29], leading to a poor outcomes of patients. Besides, COPD is also associated with CVD [29], and there were 1241 (27%) patients coexisting with CVD in our study. A variety of complex factors lead to poor nutrition in COPD patients. Clinically, we should pay more attention to the nutritional status of AECOPD patients, improving nutritional status as early as possible, maintaining muscle mass, angainst systemic inflammatory response to improve outcome.

Inflammatory response is aggravated in AECOPD. Values of anti-inflammatory biomarkers (leukocyte, neutrophil and monocyte) were abnormal in AECOPD patients unavoidablely, moreover, some studies regarded NLR, PLR and LMR as indicators of systemic inflammatory response, and further studies found higher NLR, PLR and LMR were associated with outcome of AECOPD patients [10, 11, 30], but were limited by small sample sizes. Our univariate study found AECOPD patients who died had higher NLR and PLR, and lower LMR. NLR and LMR showed statistical differences between survivors and non-survivors, however, finding of association between LMR and outcome of AECOPD patients showed contrary to previous studies. Overall, these inconsistent results of association between outcomes of AECOPD patients and indicators (PLR and LMR) are needed to be further studied.

This study has some strengths. First, this is a study based on a large sample. Second, we used LASSO regression and multivariate Cox regression analysis to screen for possible predictors and risk factors which prevent overfitting. Third, our model uses 3 predictors which are easily acquired and simplify the assess process, is of great value for clinical reference and use. Finally, we found the NLR and LMR were indeed associated with bad outcome of AECOPD patients through a large sample study.

This study has some limitations. Because of this is a retrospective, single-center study, some biases were inevitable. Firstly, Due to the extensive time span covered by the data extracted from the database (2012.01.01-2022.12.31), there were missing data for some early admission patients, with many serum indicator variables having missing values > 10%. As multiple imputation was not feasible to address this issue, we were compelled to exclude the data of these patients. This limitation may have had some impact on the study outcomes, a factor we acknowledge and prudently consider when analyzing and interpreting the research findings. Despite these constraints, we have made diligent efforts to ensure the reliability and accuracy of the study conclusions, recognizing the need for more meticulous handling of potential challenges in data collection processes in future research endeavors. Secondly, the low death rate raises concerns about the stability of the Cox regression model, to address this vital concern, during model evaluation, we employed rigorous cross-validation and resampling techniques to ensure the stability and generalizability of the model. And despite the limited number of outcome events, our study results demonstrated consistently high performance across different evaluation metrics such as C-indexes and AUC. This suggests that even in the context of a low event rate, our model can effectively differentiate patient. We also conducted an assessment of the model’s confidence intervals to ensure the reliability and stability of the results. Lastly, some important records lacked such as smoking history and pulmonary functions which are closely related to outcomes of AECOPD patients.

## Conclusions

Coexisting arrhythmia and chronic kidney disease, lower hemoglobin and albumin values, requiring oxygen therapy, systemic corticosteroids, antibiotics and IMV were associated with lower survival probability of AECOPD patients. We established a nomogram based on 3 predictors (coexisting arrhythmia, IMV usage and lower serum albumin values) for predicting the survival probability of AECOPD patients and the nomogram showed good performance.

### Electronic supplementary material

Below is the link to the electronic supplementary material.

Supplementary Material 1

Supplementary Material 2

Supplementary Material 3

Supplementary Material 4

## Data Availability

The datasets generated and analyzed during the current study are available from the corresponding author on reasonable request.

## Abbreviations

AECOPD: Acute exacerbation of chronic obstructive pulmonary disease
AUC: Area under the ROC curve
COPD: Chronic obstructive pulmonary disease
CKD: Chronic kidney disease
CVD: Cardiovascular disease
CI: Confidence interval
DCA: Decision curve analysis
FEVI: Forced expiratory volume in one-second
FVC: Forced vital capacity
ICU-saty: Intensive care unit stay
IMV: Invasive mechanical ventilation
LMR: Lymphocyte -to-monocyte ratio
LASSO: Least absolute shrinkage and selection operator
MV: Mechanical ventilation
NIMV: Noninvasive mechanical ventilation
NLR: Neutrophil-to-Lymphocyte ratio
PaO2: Aerial partial pressure of O2
PaCO2: Arterial partial pressure of CO2
PLR: Platelet-to-lymphocyte ratio
ROC: Receiver operating characteristic
SpO2: Arterial oxygen saturation
SD: Standard deviation
