# Supplementary figures and images for: Development and validation of a nomogram for assessing survival in acute exacerbation of chronic obstructive pulmonary disease patients
Source: BMC Pulm Med. 2024 Jun 19;24:287. doi: 10.1186/s12890-024-03091-w (PMC11186077; doi:10.1186/s12890-024-03091-w)

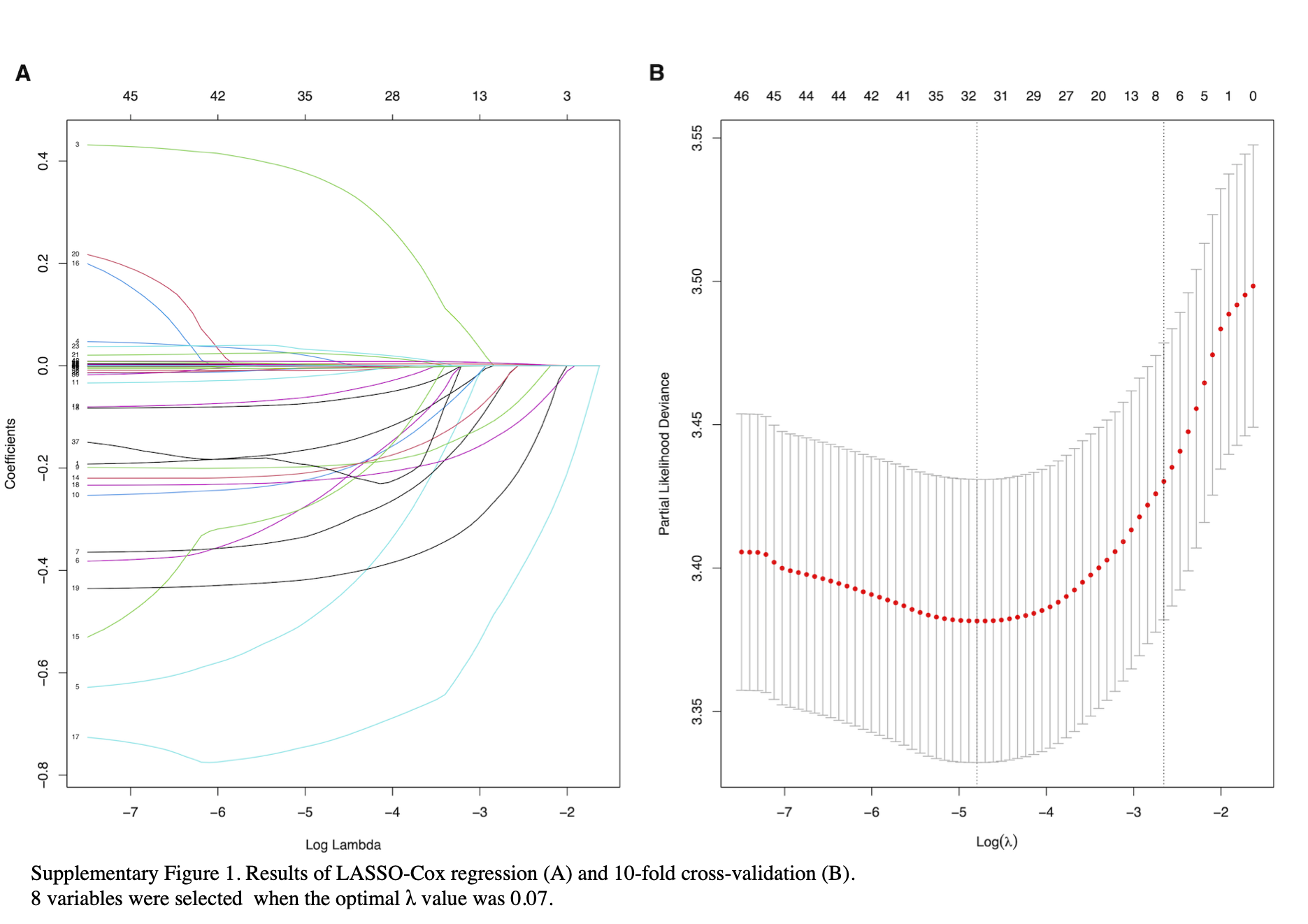

Supplement: Supplementary file 1 — Supplementary Material 1 [file 12890_2024_3091_MOESM1_ESM.tiff]

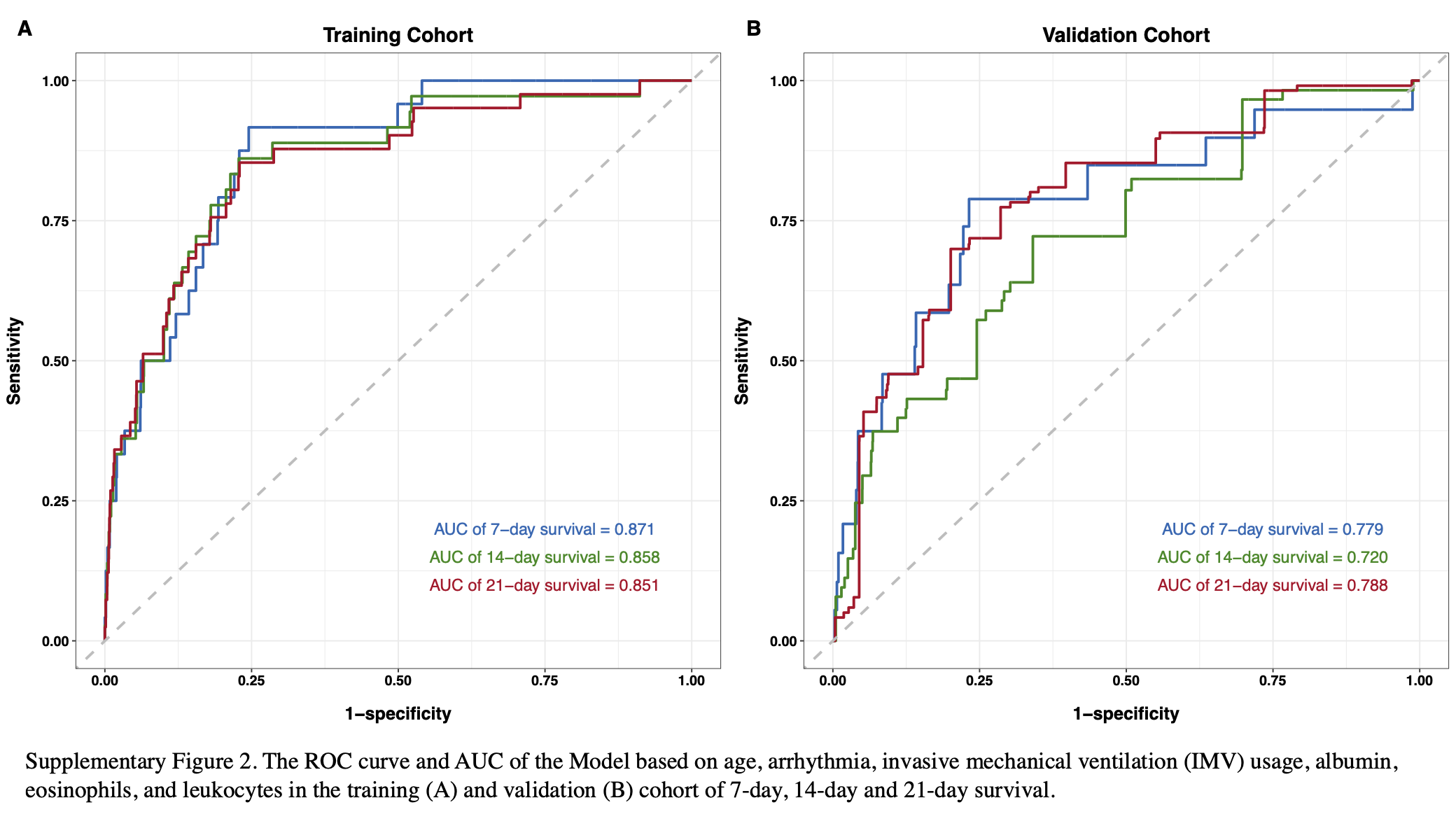

Supplement: Supplementary file 2 — Supplementary Material 2 [file 12890_2024_3091_MOESM2_ESM.tiff]

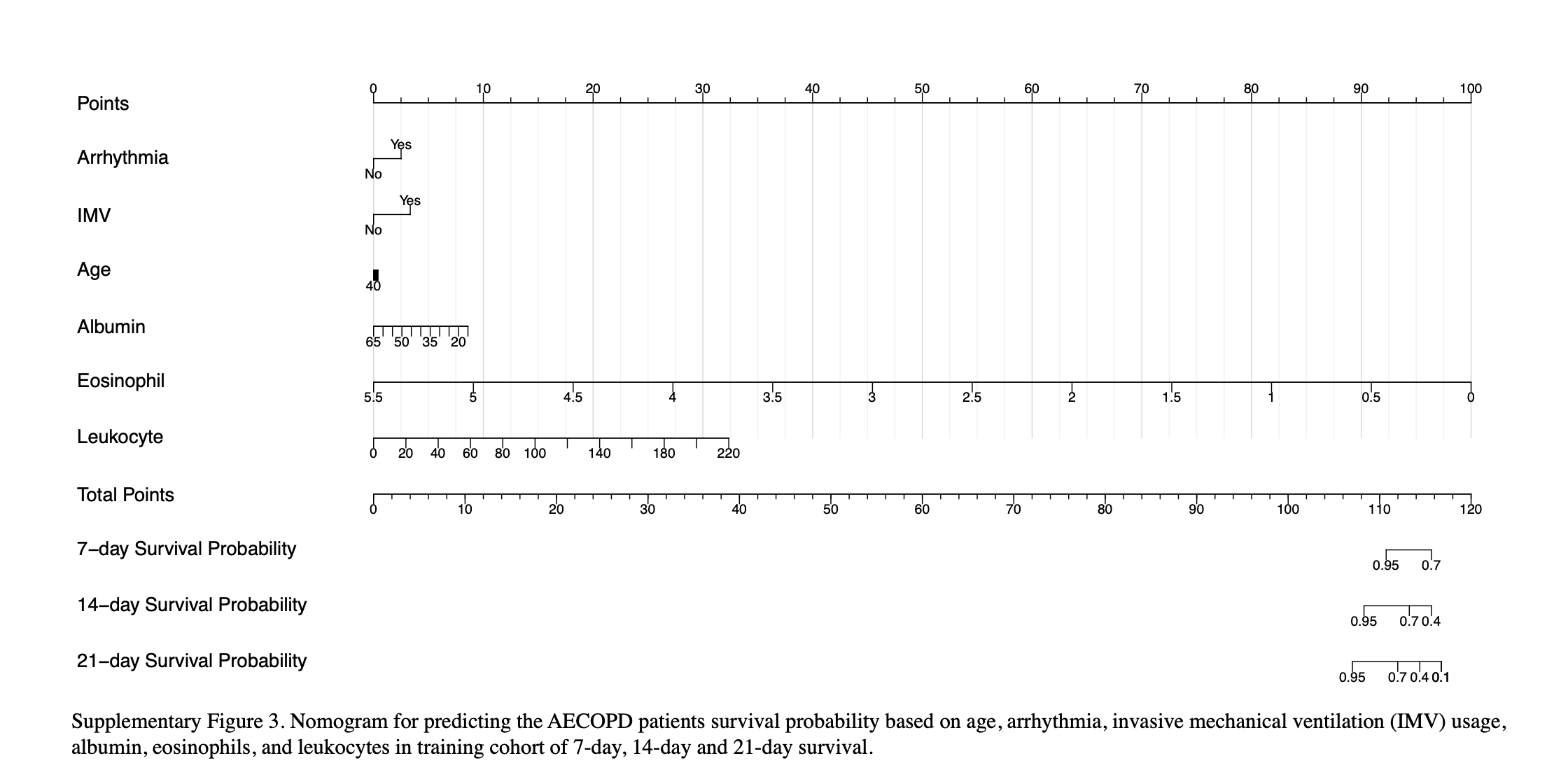

Supplement: Supplementary file 3 — Supplementary Material 3 [file 12890_2024_3091_MOESM3_ESM.tiff]
