# Supplementary material for: Development and validation of a nomogram for assessing survival in acute exacerbation of chronic obstructive pulmonary disease patients
Source: BMC Pulm Med. 2024 Jun 19;24:287. doi: 10.1186/s12890-024-03091-w (PMC11186077; doi:10.1186/s12890-024-03091-w)
Supplement: Supplementary file 4 — Supplementary Material 4 [file 12890_2024_3091_MOESM4_ESM.docx]

Supplementary Table 1.  C-index and AUC of indicators for predicting the survival in AECOPD patients.

|  | |  | | Training Cohort | | | | | |  | | Validation Cohort | | | | | |
| --- | --- | --- | --- | --- | --- | --- | --- | --- | --- | --- | --- | --- | --- | --- | --- | --- | --- |
|  | |  | | AUC | | | | | |  | | AUC | | | | | |
| Models | | C-index | | 7-day | | 14-day | | 21-day | | C-index | | 7-day | | 14-day | | 21-day | |
| Arrhythmia | | 0.517 | | 0.662 | | 0.665 | | 0.671 | | 0.431 | | 0.659 | | 0.632 | | 0.637 |  |
| IMV | | 0.528 | | 0.649 | | 0.638 | | 0.644 | | 0.411 | | 0.660 | | 0.658 | | 0.680 |  |
| Albumin | | 0.583 | | 0.721 | | 0.692 | | 0.691 | | 0.523 | | 0.731 | | 0.758 | | 0.584 |  |
| Age | | 0.116 | | 0.486 | | 0.551 | | 0.588 | | 0.246 | | 0.607 | | 0.428 | | 0.492 |  |
| Eosinophil | | 0.435 | | 0.615 | | 0.643 | | 0.617 | | 0.378 | | 0.624 | | 0.635 | | 0.539 |  |
| Leukocyte | | 0.137 | | 0.570 | | 0.557 | | 0.562 | | 0.130 | | 0.531 | | 0.532 | | 0.436 |  |
| Arrhythmia + IMV + Albumin | | 0.816 | | 0.825 | | 0.807 | | 0.825 | | 0.814 | | 0.796 | | 0.831 | | 0.841 |  |
| Arrhythmia + IMV + Age + Albumin + Eosinophil + Leukocyte | | 0.719 | | 0.871 | | 0.858 | | 0.851 | | 0.708 | | 0.779 | | 0.720 | | 0.788 |  |

Supplementary Table 1. C-index and AUC of indicators for predicting the survival in AECOPD patients for 7-day, 14-day and 21-day in both training and validation cohorts.

Abbreviations: AECOPD: acute exacerbation of chronic obstructive pulmonary disease; IMV: invasive mechanical ventilation; AUC: area under the ROC curve.
